# Isolation of a monoclonal antibody from a phage display library binding the rhesus macaque MHC class I allomorph Mamu-A1*001

**DOI:** 10.1371/journal.pone.0179039

**Published:** 2017-07-18

**Authors:** Nathan Holman, Jason T. Weinfurter, Trevor R. Harsla, Roger W. Wiseman, Aaron J. Belli, Anthony J. Michaels, Keith A. Reimann, Robert I. DeMars, Matthew R. Reynolds

**Affiliations:** 1 Department of Pathology and Laboratory Medicine, University of Wisconsin-Madison, Madison, Wisconsin, United States of America; 2 Wisconsin National Primate Research Center, University of Wisconsin-Madison, Madison, Wisconsin, United States of America; 3 MassBiologics, University of Massachusetts Medical School, Boston, Massachusetts, United States of America; US Naval Research Laboratory, UNITED STATES

## Abstract

Monoclonal antibodies that bind to human leukocyte antigen (HLA) are useful tools for HLA-typing, tracking donor-recipient chimerisms after bone marrow transplants, and characterizing specific major histocompatibility complexes (MHC) on cell surfaces. Unfortunately, equivalent reagents are not available for rhesus macaques, which are commonly used animal as models in organ transplant and infectious disease research. To address this deficiency, we isolated an antibody that recognizes the common Indian rhesus macaque MHC class I molecule, Mamu-A1*001. We induced Mamu-A1*001-binding antibodies by alloimmunizing a female Mamu-A1*001-negative rhesus macaque with peripheral blood mononuclear cells (PBMC) from a male Mamu-A1*001-positive donor. A Fab phage display library was constructed with PBMC from the alloimmunized macaque and panned to isolate an antibody that binds to Mamu-A1*001 but not to other common rhesus macaque MHC class I molecules. The isolated antibody distinguishes PBMC from Mamu-A1*001-positive and -negative macaques. Additionally, the Mamu-A1*001-specific antibody binds the cynomolgus macaque MHC class I ortholog Mafa-A1*001:01 but not variants Mafa-A1*001:02/03, indicating a high degree of binding specificity. The Mamu-A1*001-specific antibody will be useful for identifying Mamu-A1*001-positive rhesus macaques, for detecting Mamu-A1*001-positive cells in populations of Mamu-A1*001-negative cells, and for examining disease processes that alter expression of Mamu-A1*001 on cell surfaces. Moreover, the alloimmunization process we describe will be useful for isolating additional MHC allomorph-specific monoclonal antibodies or antibodies against other polymorphic host proteins which are difficult to isolate with traditional technologies.

## Introduction

Major histocompatibility complex (MHC) class I and II molecules are present in all vertebrate animals and serve an important function in adaptive cellular immune responses. They bind pathogen-derived peptides and present them on cell surfaces to CD8+ and CD4+ T cells respectively. The genes encoding MHC molecules are highly polymorphic and encode amino acid differences that affect peptide binding and T-cell recognition [1]. These subtle differences among MHC molecules can induce potent immune responses when individuals are exposed to allogeneic cells. For example, anti-human leukocyte antigen (HLA) antibodies naturally develop following exposure to fetal cells during pregnancy [2, 3], to organ transplants [4], and to multiple blood transfusions [5]. The anti-HLA antibodies raised in these individuals recognize epitopes specific to a few individual or small subsets of MHC allotypes [6, 7]. This is due to the elimination of pan-reactive B-cell receptors recognizing “self” epitopes shared by all MHC molecules during B-cell education [8]. Until recently, HLA phenotyping was commonly performed using serum from multiparous women who naturally developed anti-paternal MHC antibodies during the course of pregnancy [9]. However, the multi-specificity, low-titer, and restricted availability of defined serum limited the usefulness of anti-HLA antisera for other applications.

Advances in immortalizing human B cells and the development of phage-display technologies allowed for the isolation of anti-HLA monocolonal antibodies from allosensitized individuals [10, 11]. The anti-HLA antibodies have proven to be valuable reagents for investigating MHC molecules themselves and their role in disease processes. For example, the HLA-A2-specific antibody has been used to purify HLA-A2 molecules for the purpose of characterizing HLA-A2 bound peptides [12, 13], for confirming HLA-A2 restriction of epitope-specific CD8+ T-cell responses [14, 15], and for examining HLA-A2 cell surface downregulation by the HIV Nef protein [16, 17]. Additionally, anti-HLA antibodies are used to monitor donor and recipient cell chimerism after hematopoietic stem cell transplants [18, 19]. These studies demonstrate the utility of MHC allomorph-specific monoclonal antibodies in different areas of medical research.

Rhesus macaques are important animal models for organ transplantion and infectious disease research [20–24]. Understanding the role MHC molecules play in these models may lead to improved clinical treatment in humans. The genetic organization of the MHC is highly conserved between macaques and humans [25] and our understanding of macaque MHC genetics has significantly expanded over the past 15 years [25–28]. These advances have facilitated the use of MHC-defined macaques in pre-clinical studies. However, monoclonal antibodies specific for macaque MHC have not been available for MHC screening, for *in vitro* assays or for measuring chimerism.

The study described herein addresses this deficiency. We aimed to isolate a monoclonal antibody specific for the rhesus macaque MHC class I molecule Mamu-A1*001. We took advantage of the fact that multiparous females often make MHC antibodies directed against non-framework epitopes after multiple pregnancies and would likely be sensitized to our MHC allomorph of interest. We stimulated the production of Mamu-A1*001-binding antibodies in a multiparous Mamu-A1*001-negative macaque by alloimmunizing her with PBMC from a Mamu-A1*001-positive donor. Mamu-A1*001-binding antibodies were clearly detectable in the serum one week after the first immunization. To isolate a monoclonal antibody, we constructed a Fab phage display library using lymphocytes isolated from the alloimmunized animal and panned the library for Mamu-A1*001-specific antibodies. An antibody was isolated that binds Mamu-A1*001 but not other common Mamu-A or -B allomorphs. Additionally, the anti-Mamu-A1*001 antibody bound to the cynomolgus macaque MHC class I ortholog Mafa-A1*001:01 but not variants Mafa-A1*001:02/03. This antibody has potential utility in screening rhesus or cynomolgus macaques for Mamu-A1*001 or Mafa-A1*001:01 respectively, tracking chimerism after hematopoietic stem cell transplants, or investigating disease processes in these animal models. The approach we describe here may also be effective in isolating monoclonal antibodies against other polymporphic proteins.

## Materials and methods

### Ethics statement and animal care

Rhesus macaques (Macaca mulatta) used in this study were cared for by the staff at the Wisconsin National Primate Research Center according to regulations and guidelines of the University of Wisconsin Institutional Animal Care and Use Committee, which approved this study (protocol g00695) in accordance with recommendations of the Weatherall report and according to the principles described in the National Research Council’s Guide for the Care and Use of Laboratory Animals. Per Animal Wellfare Approved regulations, all animals were housed in enclosures with at least 4.3, 6.0, or 8.0 sq. ft. of floor space, measuring 30, 32, or 36 inches high, and containing a tubular PVC or stainless steel perch. Each individual enclosure was equipped with a horizontal or vertical sliding door, an automatic water lixit, and a stainless-steel feed hopper. All animals were fed using a nutritional plan based on recommendations published by the National Research Council. Twice daily the macaques on the described study were fed a fixed formula, extruded dry diet (2050 Teklad Global 20% Protein Primate Diet) with adequate carbohydrate, energy, fat, fiber (10%), mineral, protein, and vitamin content. Feeding strategies were individually tailored to the age and physical condition of the experimental subjects. Dry diets were supplemented with fruits, vegetables, and other edible objects (e.g., nuts, cereals, seed mixtures, yogurt, peanut butter, popcorn, marshmallows, etc.) to provide variety to the diet and to inspire species-specific behaviors such as foraging. To further promote psychological well-being, animals were provided with food enrichment, human-to-monkey interaction, structural enrichment, and manipulanda. Environmental enrichment objects were selected to minimize chances of pathogen transmission from one animal to another and from animals to care staff. While on study, all animals were evaluated by trained animal care staff at least twice each day for signs of pain, distress, and illness by observing appetite, stool quality, activity level, and physical condition. Animals abnormally presenting for any of these clinical parameters were provided appropriate care by attending veterinarians. Prior to all experimental procedures, animals were sedated using ketamine anesthesia, which was reversed at the conclusion of a procedure using atipamezole. Animals were monitored regularly until fully recovered from anesthesia. Animals were returned to the colony at the conclusion of this study.

### MHC genotyping of macaques

MHC class I sequences were determined by deep sequencing as previously described [29]. Briefly, genomic DNAs isolated from frozen whole blood samples were used as templates for PCR with a pair of primers that bind highly conserved sequences flanking the highly polymorphic peptide binding domain encoded by exon 2 of class I loci. After cleanup and pooling, these amplicons were sequenced on an Illumina MiSeq instrument and the resulting sequence reads were mapped against a custom database of rhesus macaque class I sequences.

### Alloimmunizations

We adapted an immunization regimen used to treat women for recurrent spontaneous abortions to generate MHC allomorph-binding antibodies in macaques [30, 31]. A Mamu-A1*001-negative rhesus macaque received three immunizations three weeks apart with PBMC freshly isolated from a Mamu-A1*001-positive rhesus macaque. Each immunization consisted of up to 40 million PBMC suspended in PBS containing 15U/ml heparin divided as follows: ¼ of the cells administered intravenously and the remaining ¾ of the cells divided equally between three intradermal sites on the forearm. Blood and serum was collected one-week after each immunization to construct the phage display library and screen for MHC-binding antibodies.

### Screening sera for Mamu-A1*001-binding antibodies

MHC gene transferents, a human MHC class I-deficient, Epstein-Barr virus-transformed B lymphoblastoid cell line (721.221) transfected with rhesus macaque MHC class I molecules [32, 33], and the parental MHC-class I null cell line (721.221) were incubated for 1 hour at 37°C with 20 μl of 1:20 diluted serum. The mouse anti-human MHC class I antibody (clone W6/32; Fisher Scientific) and non-specific mouse IgG1 (BD Biosciences) were included as positive and negative controls, respectively. Cell-bound antibodies were detected with anti-macaque IgG FITC (1B3; NIH Nonhuman Primate Reagent Resource) or goat anti-mouse Ig FITC (polyclonal; BD Biosciences) by incubating antibody-exposed cells for 30 minutes at room temperature. The cells were then fixed with 2% paraformaldehyde and stored at 4°C until being acquired on a LSR II flow cytometer (Becton Dickinson). The data was analyzed using FlowJo software (Tree Star).

### Construction of the Fab library

The Fab library was constructed as previously described in *Phage Display*: *A Laboratory Manual* by Barbas *et al* [34] using the pComb3X system, rhesus macaque-specific primers and conditions detailed in Kuwata *et al* [35]. The pComb3XSS phagemid was obtained from the Office of Technology Development at the Scripps Research Institute in La Jolla, CA. Briefly, total RNA was prepared from PBMC isolated 1–2 weeks post-immunization using an RNeasy Mini Kit (Qiagen) and used for first-strand cDNA synthesis with oligo(dT)_20_ primers and SuperScript III Reverse Transcriptase (Invitrogen) per the manufacturer’s instructions. Three rounds of overlap extension PCR were performed to amplify and join constant and variable regions of the immunoglobulin heavy- and light (κ and λ) chains into Fab fragments. All PCR reactions were carried out with Platinum Taq DNA polymerase High Fidelity (Thermo Fisher Scientific) and purified from agarose gels using the QIAquick Gel Extraction Kit (Qiagen). To complete the phage library, the Fab DNA fragments were ligated overnight into *sfiI*- digested (New England Biolabs) pComb3X and transformed into *E*. *coli* XL1-Blue (Stratagene) by electroporation. Transformed bacteria were inoculated into SOC medium (Thermo Fisher Scientific), incubated for 1 hour at 37°C, and grown overnight at 37°C on “Circlegrow” (MP Biomedicals) agar plates containing 100μg/ml ampicillin. Transformed cultures were grown at 37°C in 2xTY medium containing 100μg/ml ampicillin and 1% glucose (2xTY + 1% glu) until the OD_600_ reached 4. 2x10^11^ KM13 helper phages were added and the culture was incubated for 30 minutes at 37°C. Pelleted bacteria were suspended in 2xTY medium containing 100 μg/ml ampicillin, 50μg/ml kanamycin, plus 0.1% glucose and grown overnight at 30°C. Library phages were precipitated from the culture medium with 20% (w/v) polyethylene glycol (PEG) 8000/ 2.5M NaCl. The size of the library was estimated by infecting *E*. *coli* with serially diluted phage and counting the number of colony-forming units.

### Panning for Mamu-A1*001-binding phages

The first four rounds of panning were performed by mixing 8μg of biotinylated Mamu-A1*001 monomer acquired from either the NIH or Wisconsin National Primate Research Center Tetramer Cores with 10^10^ phage and incubating for 1 hour at room temperature. The MHC-binding phages were captured by adding streptavidin-coupled magnetic beads (Invitrogen) suspended in “PBS Tween” containing 4% fetal bovine serum (FBS) and 1% Tween 20 and incubated for 30 minutes at room temperature. Phage-MHC-streptavidin bead complexes were washed ten times with PBS Tween by applying the tube containing the suspended complexes to an external magnet (Invitrogen) and discarding the unretained beads in the supernatant. MHC-binding phages were eluted from the bead complexes by incubating with 0.1M glycine at room temperature for 15 minutes. The mixture was magnet precipitated and neutralized with 1M Tris-HCL (pH = 7.4). The released phages were amplified in *E*.*coli* TG1 (Source BioScience). A negative selection to remove pan-MHC binding phages was performed prior to the fourth round of panning by incubating with biotinylated Mamu-A1*002 monomers. The supernatant from this step was used to complete the panning process with Mamu-A1*001 monomers.

Two additional rounds of panning were performed with MHC class I transferents. Prior to both rounds, negative selection was performed by incubating phages for 30 minutes at room temperature with transferents expressing Mamu-B*008. The cells were then pelleted and the supernatant collected to pan with Mamu-A1*001 transferents. The mixture of phages and cells were incubated for 30 minutes at room temperature. The cells were washed ten times with FACS buffer. Cell-bound phage was eluted using 100mM triethylamine and neutralized with 1M Tris-HCL.

### ELISA to detect Mamu-A1*001-specific phages

MHC-coated 96-well plates were prepared by incubating pre-blocked streptavidin-coated plates (Pierce) with biotinylated rhesus macaque MHC class I monomers (20 μg/ml) and incubating for one hour at room temperature. The wells were then washed three times with PBS containing 2% Tween 20 (T-PBS).

*E*.*coli* TG1 was infected with phages, grown overnight at 37°C on TYE plates containing 100 μg/ml ampicillin and 1% glucose. Isolated bacterial colonies were grown overnight in 96-deep-well plates (Thermo Fisher Scientific) in 2xTY + 1% glu at 37°C while shaking. 10μl from each well were inoculated into a well on a second 96-deep-well plate containing 2xTY+1% glu and incubated for 3 hours at 37°C. 100μl of 2xTY+1% glu medium containing 10^9^ KM13 helper phage was added to each well and incubated for one hour at 37°C. The bacteria were resuspended in 200μl of 2xTY containing 100μg/ml ampicillin, 0.1% glucose, and 50 μg/ml kanamycin and were grown overnight at 30°C. After pelleting, 100μl of each phage-containing supernatant was added to the prepared MHC-coated ELISA plates and incubated for 90 minutes at room temperature. The mouse pan-MHC class I-binding antibody W6/32 was used as a positive control and medium alone was used as a negative control. The plate was washed three times with T-PBS. Bound phages were detected with a 1:5,000 dilution of horseradish peroxidase (HRP)-conjugated anti-M13 antibody (GE Healthcare Life Sciences catalog # 27-9421-01) in PBS containing 4% bovine serum albumin and incubated for 40 minutes at room temperature. The mouse anti-MHC antibody was detected with goat anti-mouse IgG-HRP (Thermo Fisher Scientific catalog # AHI0704). The plates were washed three times with T-PBS. The colorimetric substrate TMB (Thermo Fisher Scientific) was added to each well and incubated at room temperature for 2–30 minutes per manufacturer’s instructions. The reaction was stopped with 50μl of 0.18 M sulphuric acid and read on a GloMax Multi plate reader (Promega) at an OD_450_.

### Anti-Mamu-A1*001 Fab production

*E*.*coli* XL1-Blue was infected with a Mamu-A1*001-binding phage clone by inoculating an individual bacterial colony into 10ml of Super broth (10g MOPS (3-[*N*-morpholino]-propanesulfonic acid), 30g tryptone, 20g yeast extract, 1L distilled water) containing 100ug/ml ampicillin plus 10ug/ml tetracycline and incubating at 37°C overnight. The overnight culture was added to 1L of Super broth and incubated at 37°C. After 8 hours, Fab production was induced by adding 10ml of 0.1M isopropyl β-D-1-thiogalactopyranoside (IPTG; Sigma-Aldrich) and incubated overnight at 37°C. The bacteria were pelleted and suspended in 20ml of PBS containing 200μM phenylmethanesulfonyl fluoride (PMSF; Sigma-Aldrich). The bacterial suspension was sonicated using a Branson Sonifier 250 in an ice-water bath for 180 seconds, with 50% duty cycle, and output control set to 5. The cellular debris was removed by centrifugation.

### Detection of MHC-binding phage or Fab antibody fragments by flow cytometry

2-5x10^5^ rhesus or cynomolgus macaque PBMC or MHC transferents were incubated for 30 minutes at 4°C with 50μl of phage-containing supernatant or Fab-containing bacterial lysate. The MHC class I-specfic antibody W6/32 was used as a positive control and non-specific mouse IgG1 was used as a negative control. The cells were washed twice with FACS buffer (PBS + 2% FBS). Bound Fab fragments fused to a HA tag were detected with high affinity anti-HA-FITC (3F10; Roche Diagnostics) or bound phages were detected with anti-M13-PE (RL-PH2; Santa Cruz Biotech). The control antibodies were detected by incubation with goat anti-mouse Ig FITC or PE (BD Biosciences) for 50 minutes at room temperature. The cells were washed twice with FACS buffer, fixed with 2% paraformaldehyde (PFA) and stored at 4°C until being acquired on a LSR II flow cytometer. The data was analyzed using FlowJo software.

### Construction of a full-length anti-Mamu-A1*001 IgG molecule

The isolated anti-Mamu-A1*001 Fab antibody gene was sequenced and the heavy and light chain variable regions were cloned into pCOXW expression vector containing rhesus IgG1 and rhesus kappa constant regions. The full-length rhesus IgG1κ antibody was expressed by transient transfection of 293T cells using Lipofectamine 2000. Antibody was purified using HiTrap Protein A HP resin and then conjugated to R-phycoerythrin using thiol maleimide coupling.

### Screening whole blood to identify Mamu-A1*001 positive macaques

100μl of fresh or frozen whole blood from rhesus macaques were placed into cluster tubes (Thermo Fisher Scientific) and incubated with either the anti-Mamu-A1*001 Fab-containing bacterial lysate, MHC class I-specific antibody W6/32 (positive control), or non-specific mouse IgG1 (negative control) for 30 minutes at room temperature. The red blood cells were lysed by incubating with BD FACS Lyse solution (BD Biosciences) for 10 minutes at room temperature. The cells were washed twice with FACS buffer. Cell-bound anti-Mamu-A1*001 Fab fragments were detected with anti-HA FITC and control antibodies with goat anti-mouse Ig FITC secondary antibodies. After a 30-minute incubation at room temperature the cells were washed twice with FACS buffer, fixed with 2% paraformaldehyde (PFA) and stored at 4°C until being acquired on a LSR II flow cytometer. The data was analyzed using FlowJo software.

## Results

### Alloimmunization of a multiparous female rhesus macaque

We endeavored to generate and isolate a monoclonal antibody against the rhesus macaque MHC class I molecule Mamu-A1*001. This molecule is expressed by a high frequency of Indian-origin rhesus macaques at primate colonies in the United States [25] and Mamu-A1*001-positive macaques are commonly used in Simian Immunodeficiency Virus (SIV) infection studies. Consequently, SIV-derived CD8+ T-cell epitopes restricted by Mamu-A1*001 have been well characterized [32, 36, 37]. This has led to the development of Mamu-A1*001-specific reagents for tracking antiviral CD8+ T-cell responses including Mamu-A1*001 monomers [32, 38–40]. The availability of these reagents made Mamu-A1*001 an attractive target for isolating a rhesus macaque allomorph-specific monoclonal antibody.

**Fig 1:**
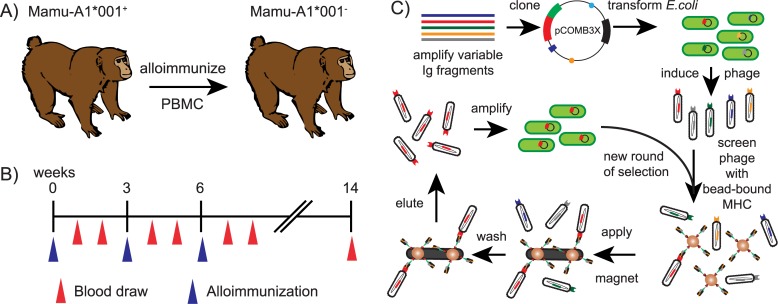
Overview of the antibody selection process. A) PBMC from a Mamu-A1*001 positive macaque was used to alloimmunize a Mamu-A1*001 negative macaque to induce Mamu-A1*001-binding antibodies. B) Timeline for the immunizations and blood draws for the alloimmunized animal. C) The panning process to isolate the Mamu-A1*001-binding antibody was initiated by amplifying immunoglobulin genes from the alloimmunized macaque and inserting into the phagemid pCOMB3X. The resulting phage library was screened with biotinylated MHC molecules bound to streptavidin-coated magnetic beads and applied to a magnet to wash away unbound phage. Bound phage was eluted and amplified in *E*.*coli* for new rounds of selection.

Multiparous female animals often develop alloreactive antibody responses to non-self-epitopes that distinguish individual MHC allomorphs rather than framework epitopes present on all MHC molecules [10]. To stimulate production of Mamu-A1*001-binding antibodies, we alloimmunized a Mamu-A1*001-negative macaque mother of multiple Mamu-A1*001-positive offspring with a lymphocyte immunotherapy regimen previously employed to treat humans for recurrent spontaneous abortions (Fig 1) [31]. In the human clinical trials, the alloimmunizations were well-tolerated and readily induced anti-MHC antibodies [30, 41]. The donor and recipient macaques were also mismatched at their MHC B loci haplotypes (Table 1).

**Table 1:** MHC haplotypes of donor and recipient rhesus macaques.

Animals	Haplotype A1	Haplotype A2	Haplotype B1	Haplotype B2
Alloimmunized female macaque	A004	A023	B001	B017c
PBMC donor macaque	A001	A001	B012a	B024a

**Fig 2:**
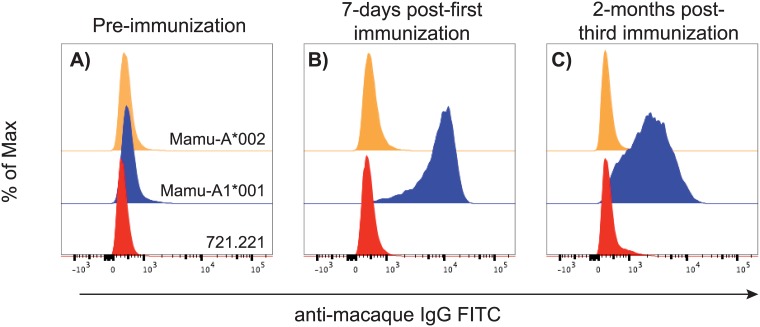
Screening serum of the alloimmunized macaque for Mamu-A1*001-binding antibodies. MHC class I transferents expressing only either Mamu-A1*001 or Mamu-A1*002 and the parental MHC class I-null cell line (721.221) were incubated with (A) pre-, (B) 7-days post-, and (C) 2-months post-immunization serum from the alloimmunized macaque. Cell-bound serum antibodies were detected with FITC-labeled anti-macaque IgG FITC.

We screened for MHC-binding antibodies in the serum of the alloimmunized macaque using MHC “transferents”, an immortalized human B-cell line expressing single MHC class I molecules in isolation [33]. Mamu-A1*001-binding antibodies were detected in the serum one week after the first PBMC alloimmunization. We observed a distinct shift in the histogram of the Mamu-A1*001 transferent with serum from the alloimmunized female in comparison to incubation with pre-immunization serum, serum incubated with non-Mamu-A1*001 transferents, or the parental MHC class I-null 721.221 cell line (Fig 2). The Mamu-A1*001-binding antibody responses were durable as they were detected in the serum two months after the third alloimmunization. Although the MHC transferent panel did not cover all potential rhesus macaque MHC molecules the results from the serum screen we performed indicated that the alloimmunized animal contained B cells that specifically recognized Mamu-A1*001.

### Construction and panning of a Fab phage display library

Isolating monoclonal antibodies from primates using conventional hybridoma techniques can be challenging due to the difficulty in isolating sufficient numbers of B cells from peripheral blood and the low efficiency of B-cell/myeloma fusions [42]. Therefore, we attempted the alternative of constructing a fragment antigen binding (Fab) phage display library from the PBMC of the alloimmunized macaque to isolate a Mamu-A1*001-specific antibody. Rhesus macaque-specific primers were used to amplify the variable regions of the immunoglobulin genes as previously described by Kuwata et al [35]. The amplified Fab antibody fragments were cloned into the pComb3X phage display system [34]. The diversity of the Fab library was estimated to be 2x10^12^ different antibody molecules.

Biotinylated MHC monomers were used to initiate panning the phage library for antibody fragments that specifically bound to Mamu-A1*001. The random combinations of immunoglobulin variable fragments created during construction of the phage library had the potential to generate Fabs recognizing determinants common to all or a large subset of MHC molecules. To remove pan-reactive Fabs we performed negative selections with Mamu-A1*002 monomers to adsorb phages binding shared MHC epitopes or biotin. After each round of panning we observed increased numbers of transduced *E*.*coli*, indicating enrichment for phages that bound to Mamu-A1*001. We isolated 96 phage clones after the fourth round of panning and tested their specificity against Mamu-A1*001. Mamu-B*008 monomers were used as a negative control for non-specific binding. Twelve of the 96 clones bound to Mamu-A1*001 but, disappointingly, also bound to Mamu-B*008, indicating that negative selection with Mamu-A1*002 had not removed all phages reactive with other MHC allomorphs. Using the phages from the fourth round of panning, two additional rounds of cell-based panning with Mamu-A1*001 and Mamu-B*008 transferents were performed. The phages were pre-incubated with Mamu-B*008 transferents to remove unwanted phage prior to positively selecting for those binding Mamu-A1*001.

**Fig 3:**
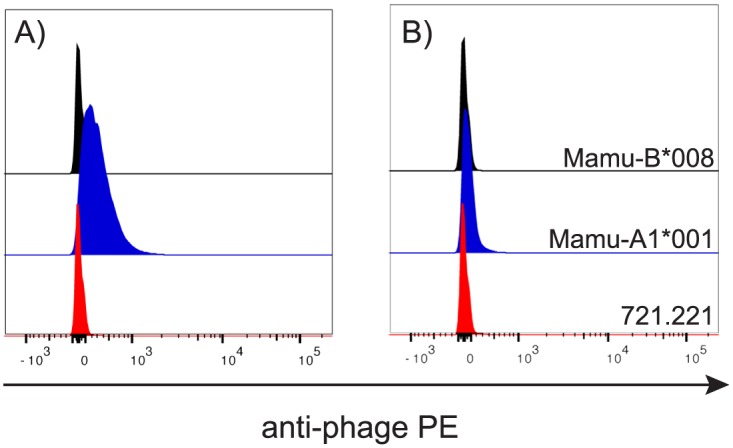
Testing the specificity of Mamu-A1*001-panned phages with MHC class I transferents. Mamu-A1*001-panned phage clones were incubated with the MHC class I-null cell line 721.221 and with MHC class I transferents expressing Mamu-A1*001 or Mamu-B*008. Cell-bound phages were detected with PE-labeled anti-M13 phage antibody. Representative data from the phage clones that did (A) or did not (B) preferentially bind to Mamu-A1*001 transferents are shown.

After the sixth round of library panning, 96 new phages were cloned and tested for their ability to discriminated between Mamu-A1*001 and -B*008 in a flow cytometry-based assay. Six of the phage clones bound to Mamu-A1*001 but not Mamu-B*008 (Fig 3), signaling that the last two rounds of panning eliminated cross reactive clones and enriched for those specific for Mamu-A1*001. Additionally, this suggested that we had isolated phage clones that could, at a minimum, distinguish Mamu-A1*001 from Mamu-A1*002 and Mamu-B*008. However, further analysis was necessary to fully characterize the MHC allomorph-binding specificity.

### Screening Mamu-A1*001-binding Fabs against whole cells

In order to be a useful reagent, the Mamu-A1*001-binding antibody needs to distinguish Mamu-A1*001 from a broad variety of rhesus macaque MHC class I molecules. Although we were encouraged by the specificity of the phages binding to Mamu-A1*001 but not Mamu-B*008 transferents, the intensity of the staining was low in comparison to the anti-MHC monoclonal antibody positive control (data not shown). In order to gain better separation between positive and negative cell populations, we produced in bulk the Fab molecule from phage clone number 12 that had the strongest Mamu-A1*001-binding characteristics. Therefore, hereafter we refer to this Fab fragment as P12. P12 was produced in the periplasm of transformed *E*.*coli* and collected by lysing the bacteria. As part of the pComb3X vector system, P12 was fused to human influenza hemagglutinin (HA) tag for detection with secondary antibodies.

**Fig 4:**
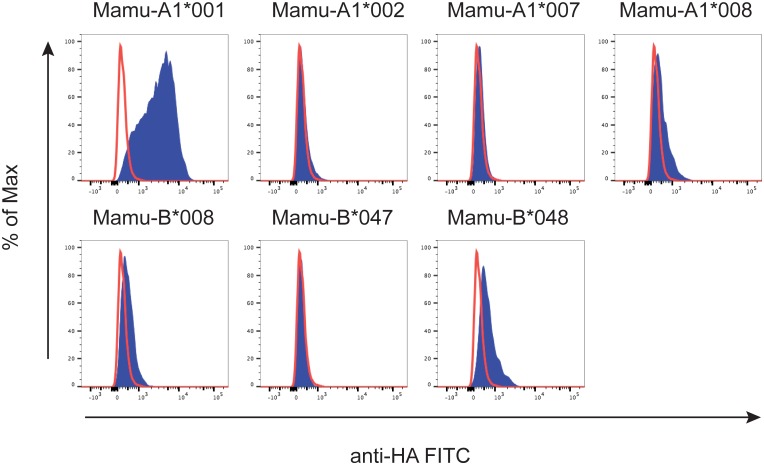
Testing the specificity of P12 against a panel of MHC class I transferents. P12 was incubated with MHC class I transferents expressing common Mamu-A and Mamu-B alleles (blue traces) and the MHC class I-null cell line 721.221 (red traces). Cell-bound Fab fragments were detected with FITC-labeled anti-HA antibody.

Initial screening of P12 was performed with a panel of eleven MHC transferents expressing just one of five Mamu-A or six -B allomorphs. Fig 4 shows that P12 substantially bound to the Mamu-A1*001 transferent, but negligibly to other MHC transferents. This indicated for the first time that the isolated Fab fragment was likely specific for Mamu-A1*001 and could distinguish between Mamu-A1*001-positive and -negative cell populations by flow cytometry.

**Fig 5:**
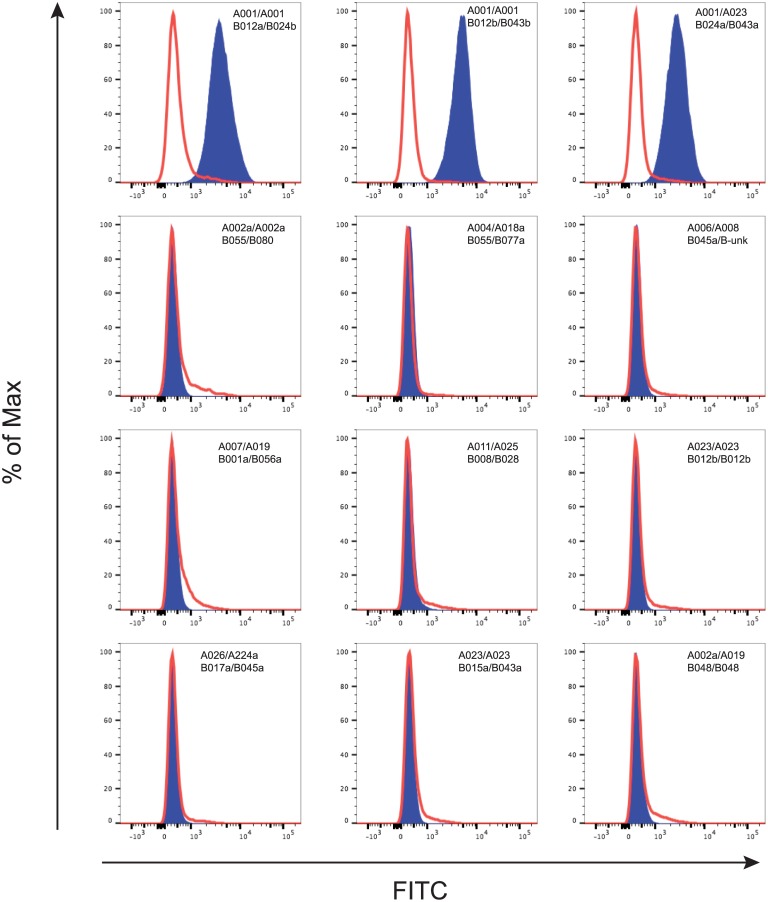
Assessing the specificity of P12 against a panel of PBMC. PBMC from Mamu-A1*001-positive (top row) and -negative (bottom three rows) rhesus macaques were stained with P12 (blue traces) or non-specific mouse Ig (red traces). Cell-bound P12 was detected with FITC-labeled anti-HA antibody and control antibody with FITC-labeled goat anti-mouse Ig. The MHC haplotypes from which the PBMC were isolated are provided in the upper right corner or each histogram.

Encouraged by the apparent specificity and enhanced binding of P12 molecules to Mamu-A1*001 we tested them against a panel of MHC-diverse Indian rhesus macaque PBMC. We identified nine Mamu-A1*001-negative macaques expressing common MHC-A and -B haplotypes. These cells provided a more rigorous test of P12 specificity since they expressed combinations of diverse MHC molecules and not just single MHC class I allomorphs expressed in isolation on MHC transferents. PBMC isolated from the nine Mamu-A1*001 negative macaques, along with PBMC from three Mamu-A1*001-positive macaques, were stained with P12 (Fig 5). The staining clearly demonstrated that the Fab only bound PBMC from animals expressing Mamu-A1*001 and not any of the MHC present on the 12 MHC-A or 14 –B haplotypes expressed by the Mamu-A1*001-negative macaques. Although it is formally possible that P12 cross-reacts with relatively rare MHC allomorphs present in captive Indian rhesus macaques, our results suggest that P12 can distinguish between cells expressing Mamu-A1*001 and cells expressing any of many other common Indian rhesus macaque MHC class I allomorphs. Of note, we did not test P12 against cells from Chinese-origin rhesus macaques. Therefore, we do not know whether P12 will bind to Mamu-A1*001 variants expressed by Chinese rhesus macaques or will bind to additional MHC allomorphs expressed by these macaques.

### Detecting small frequencies of Mamu-A1*001-positive cells

**Fig 6:**
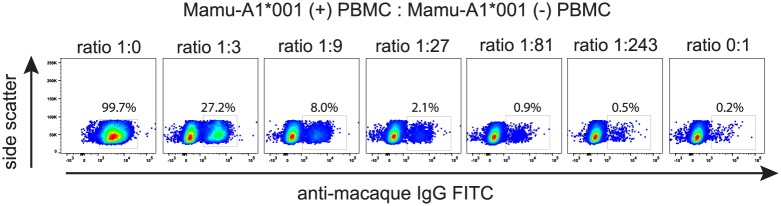
Detection of rare Mamu-A1*001-positive cells among predominantly Mamu-A1*001-negative cells. Mixtures of serial 1:3 dilutions of Mamu-A1*001-positive PBMC to Mamu-A1*001-negative PBMC were exposed to P12 and detected with FITC-labeled anti-HA antibody by means of flow cytometry. The percentages represent the frequencies of Mamu-A1*001-positive lymphocytes detected in each sample.

A potential application for an anti-Mamu-A1*001 antibody is the specific detection of infrequent Mamu-A1*001-positive cells among a preponderance of Mamu-A1*001-negative cells, as in detecting chimerisms in macaques after hematopoietic stem cell transplants. To test P12 for this purpose we made artificial mixtures of Mamu-A1*001-positive and -negative PBMC. Five serial 1:3 dilutions of Mamu-A1*001-positive cells with Mamu-A1*001-negative cells were stained with P12. We clearly detected the presence of 0.9% (1:81 dilution) and, potentially, 0.5% (1:243 dilution) of Mamu-A1*001-positive cells among Mamu-A1*001-negative cells (Fig 6). This result indicates that P12 can be a useful tool for detecting chimerisms or for isolating infrequent Mamu-A1*001-positive cells within a population of Mamu-A1*001-negative cells.

### Identifying Mamu-A1*001-positive macaques with P12

**Fig 7:**
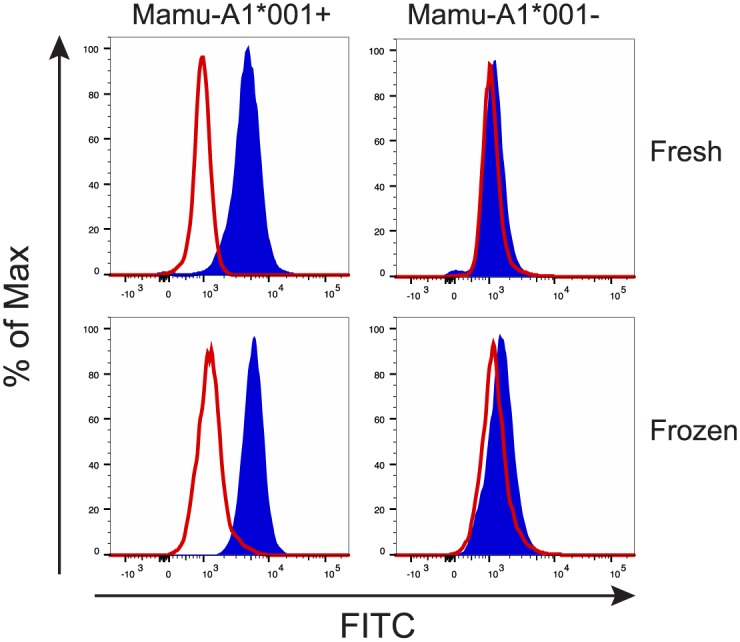
Detection of Mamu-A1*001-positive animals using whole blood and P12. Fresh or frozen whole blood from Mamu-A1*001-positive and Mamu-A1*001-negative macaques were stained with P12 (blue traces) or non-specific mouse Ig (red traces). Red blood cells were lysed, cell-bound P12 was detected with anti-HA FITC and cell-bound control antibody was detected with anti-mouse Ig FITC. Representative data from the four animals are shown.

Anti-HLA monoclonal antibodies and HLA-sensitized serum were commonly used to HLA type humans. Therefore, we sought to determine whether P12 could be used to screen for Mamu-A1*001-positive macaques. Blood from two Mamu-A1*001-positive and two negative rhesus macaques were collected and used to develop a screening assay. Since it is more practical to collect and freeze blood from large cohorts of animals over time for concurrent screening, we also tested fresh versus frozen whole blood. P12 was incubated with either fresh or thawed whole blood. Red blood cells in the samples were subsequently lysed and cell-bound P12 was detected with an anti-HA FITC antibody. P12 bound both fresh and frozen cells from Mamu-A1*001-positive animals but not negative controls (Fig 7). Although we detected similar results in the fresh and frozen samples the freeze/thaw process negatively affected lymphocyte viability. It is, therefore, possible that the freezing process may hinder the ability of P12 to accurately distinguish Mamu-A1*001-positive from -negative macaques. Inclusion of a viability dye into the staining design may help in identifying Mamu-A1*001-positive macaques, particularly when using frozen samples.

In order to test whether or not P12 could prospectively distinguish Mamu-A1*001-positive from -negative rhesus macaques we stained frozen blood samples from 48 animals. All animals were subsequently MHC genotyped, the gold standard for MHC typing, to determine their MHC haplotypes. P12 identified all of the 15 Mamu-A1*001-positive macaques, in complete concordance with those obtained by MHC genotyping. This indicates that P12 has the potential to screen animals for Mamu-A1*001 by flow cytometry. It is important to note that this cohort of animals had representatives from 14 Mamu-A and 19 Mamu-B haplotypes without any apparent cross reactivity by P12.

### P12 crossreacts with Mafa-A1*001:01 but not Mafa-A1*001:02/3 allomorphs

P12 effectively binds to Mamu-A1*001 but it lacks constant region domains important for many antibody functions. Additionally, the presence of two anti-Mamu-A1*001 Fab on the same molecule as in natural antibodies, should increase the avidity of the antibody/Mamu-A1*001 interaction. Therefore, to expand the potential utility of the anti-Mamu-A1*001 Fab we reconstituted the complete IgG molecule. As anticipated, the anti-Mamu-A1*001 IgG antibody only bound cells from Mamu-A1*001-positive animals when tested against the same panel of 12 rhesus macaque PBMC initially used to screen P12 (data not shown).

**Fig 8:**
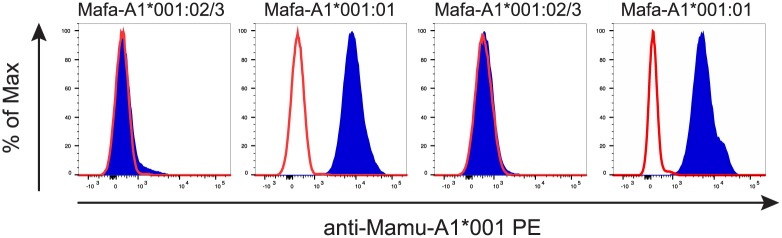
The P12 antibody differentially binds Mafa-A1*001 variants. PBMC from four cynomolgus macaques expressing the Mamu-A1*001 ortholog Mafa-A1*001 were stained with PE-labeled P12 antibody (blue traces) or left unstained (red traces). The P12 antibody only bound cells from animals expressing the Mafa-A1*001:01 allomorph but not the Mafa-A1*001:02/03 variants. The Mafa-A1*001 allele variant that each animal expressed is listed above each histogram.

Cynomolgus macaques are commonly used in infectious disease and transplant research. Analysis of MHC class I alleles in rhesus and cynomolgus macaques has revealed extensive sharing of alleles between the two species [43]. Consequently, the cynomolgus macaque MHC class I allele Mafa-A1*001 is a highly related ortholog of Mamu-A1*001. We, therefore, determined if the P12 antibody would bind to Mafa-A1*001. Cells from four Mafa-A1*001-positive cynomolgus macaques were stained with the full-length P12 antibody. Unexpectedly, the antibody only bound cells from two of the four cynomolgus macaques (Fig 8). We hypothesized that the discrepancy in antibody binding was due to the expression of different Mafa-A1*001 variants among the four macaques. Sequence-based genotyping showed that cells from the animals to which the P12 antibody bound expressed Mafa-A1*001:01, but the other two animals expressed either the Mafa-A1*001:02 or Mafa-A1*001:03 variant (the sequence-based genotyping could not distinguish between these two variants). A maximum of 11 amino acids differ between the Mafa-A1*-001:01 and the Mafa-A1*001:02 (5 amino acids) or Mafa-A1*001:03 (11 amino acids) variants. However, four of these 11 amino acid differences shared by the Mafa–A1*001:02/:03 variants are present in the alpha 1 and 2 domains, previously shown to be critical for MHC allomorph-specific binding of antibodies [44, 45].

## Discussion

We report herein the isolation from a phage display library of an antibody that specifically recognizes the common rhesus macaque MHC class I allomorph Mamu-A1*001. The Fab phage display library was constructed by amplifying immunoglobulin genes from a Mamu-A1*001-negative multiparous female rhesus macaque that had been alloimmunized with cells from a Mamu-A1*001-positive macaque. The phage library was panned using a combination of MHC monomers and MHC transferent cell lines expressing only a single MHC class I molecule. The isolated anti-Mamu-A1*001 Fab bound only to cells expressing Mamu-A1*001 and not to cells expressing other common Indian rhesus macaque MHC class I A or B alleles. We have reconstituted the complete IgG molecule and it is available to the scientific community through the Nonhuman Primate Resource Reagent Program.

Monoclonal antibodies are often isolated by immunizing mice with antigens of interest, making hybridomas by fusing B lymphocytes and myeloma cells, and screening for antigen-specific antibodies. However, this approach is difficult when isolating monoclonal antibodies specific for variants of primate polymorphic proteins since mice preferentially target xenogeneic framework determinants [10]. We overcame this hurdle by alloimmunizing a rhesus macaque with PBMC from an MHC disparate macaque to induce MHC allomorph-specific antibodies. This approach avoided induction of antibodies against common framework epitopes due to clonal deletion of self-reactive B cells. To isolate antibodies of interest we created a phage display library that captured the post-immunization immunoglobulin repertoire. The library was panned with a combination of MHC monomers and MHC transferents to isolate an anti-Mamu-A1*001-specific antibody. These methods may also be useful in isolating monoclonal antibodies against other polymorphic host proteins.

Nonhuman primates are genetically more similar to humans than small animal models. Despite their widespread use, nonhuman primates are potentially underutilized for the production and isolation of monoclonal antibodies or antibody fragments. This is in part due to the cost associated with animal husbandry, housing, and experimental procedures. However, as we describe here, macaques may provide a valuable resource in isolating antibodies targeting polymorphic cellular proteins of nonhuman primate origin. Similar approaches have previously been used to isolate antibodies from nonhuman primates against therapeutically relevant infectious disease and human antigens [46]. However, the immunogens in these studies were infectious agents or purified protein rather than allogeneic cells used in our study. There are several advantages to using nonhuman primates for isolating monoclonal antibodies specific for variants of primate polymorphic proteins. First, central tolerance established during immune development has eliminated B cells reactive to “self” determinants shared among polymorphic macaque proteins. Second, alloimmunization allows for affinity maturation and isolation of high-affinity antibodies. Third, phage-display libraries can be constructed with cells isolated from the blood or bone marrow, which can be collected without euthanasia and, thereby, minimizing animal use and preserving a valuable resource.

Indian rhesus macaques are commonly used as models for organ transplantation and infectious diseases. Identifying macaques expressing specific MHC molecules can be an essential component of experiment design since expression of particular MHC molecules may play an important role in disease progression and the success or failure of organ transplants [20, 25, 47]. For Indian rhesus macaques, next generation sequencing has become the gold standard for MHC typing since it can identify all major and minor MHC class I and II alleles. However, this approach takes specialized equipment and training to execute. The anti-Mamu-A1*001 monoclonal antibody, therefore, may be useful to researchers to perform preliminary screens for Mamu-A1*001 positive rhesus macaques. We tested the specificity of the anti-Mamu-A1*001 Fab fragments against panels of whole blood from animals expressing a diverse array of MHC molecules commonly found in Indian rhesus macaques. We only detected antibody binding to cells from Mamu-A1*001 positive macaques. The screening assay can be performed by anyone with basic experience with flow cytometry, access to macaque samples, and a flow cytometer.

Rhesus macaques are used as models for hematopoietic stem cell transplantation. A key analysis in these studies to assess engraftment or rejection is the measurement of post-transplant chimerism, or the presence of donor cells in the peripheral blood of the graft recipient. Several PCR-based chimerism assays have been adapted from human clinical assessments for use in nonhuman primate studies [48–50]. However, these analyses are time consuming and cannot distinguish between cell populations or lineages without isolating sub-populations prior to analysis. An alternative approach when MHC-mismatched transplants are performed is to track chimerism with anti-MHC antibodies by flow cytometry [18, 51]. These techniques are relatively fast and can be performed in any standard flow cytometry lab to monitor engraftment of different cell subpopulations using anti-MHC antibodies. The anti-Mamu-A1*001 antibody that we isolated will be useful in tracking Mamu-A1*001-positive cells in Mamu-A1*001-negative rhesus macaques after hematopoietic stem cell transplants. Using an artificial mixture of cells, we detected a frequency of approximately 1% Mamu-A1*001-positive cells in a population of Mamu-A1*001-negative cells. This is comparable to a previous study using a single anti-HLA antibody to detect chimeric human cells [18, 52]. The isolation of additional anti-macaque MHC class I antibodies may enhance the detection chimerisms in macaques and facilitate cell sorting of these populations for further analysis.

In this study, we demonstrate that MHC allomorph-specific monoclonal antibodies can be isolated from alloimmuninzed rhesus macaques using a phage display technology. The methods we describe do not require any specialized equipment or technical expertise not already found in most modern immunology laboratories. Therefore, this approach will likely be applicable to other model systems attempting to isolate MHC allomorph-specific antibodies. Additionally, alloimmunization with PBMC or other cell types may be useful for isolating antibodies specific for other polymorphic host proteins.

## References

[1] Trowsdale J, Knight JC. Major histocompatibility complex genomics and human disease. Annu Rev Genomics Hum Genet. 2013;14:301–323. doi: 10.1146/annurev-genom-091212-153455 23875801 10.1146/annurev-genom-091212-153455 PMC4426292

[2] PAYNE R, ROLFS MR. Fetomaternal leukocyte incompatibility. J Clin Invest. 1958;37:1756–1763. doi: 10.1172/JCI103768 13611043 10.1172/JCI103768 PMC1062862

[3] VAN ROOD JJ, EERNISSE JG, VAN LEEUWEN A. Leucocyte antibodies in sera from pregnant women. Nature. 1958;181:1735–1736. 13566127 10.1038/1811735a0

[4] Cardarelli F, Pascual M, Tolkoff-Rubin N et al Prevalence and significance of anti-HLA and donor-specific antibodies long-term after renal transplantation. Transpl Int. 2005;18:532–540. doi: 10.1111/j.1432-2277.2005.00085.x 15819801 10.1111/j.1432-2277.2005.00085.x

[5] PAYNE R. Leukocyte agglutinins in human sera; correlation between blood transfusions and their development. AMA Arch Intern Med. 1957;99:587–606. 13410165 10.1001/archinte.1957.00260040087010

[6] Duquesnoy RJ, Marrari M, Mulder A, Claas FH, Mostecki J, Balazs I. Structural aspects of human leukocyte antigen class I epitopes detected by human monoclonal antibodies. Hum Immunol. 2012;73:267–277. doi: 10.1016/j.humimm.2011.11.011 22227099 10.1016/j.humimm.2011.11.011

[7] Duquesnoy RJ. Reflections on HLA Epitope-Based Matching for Transplantation. Front Immunol. 2016;7:469 doi: 10.3389/fimmu.2016.00469 27965660 10.3389/fimmu.2016.00469 PMC5124729

[8] Wardemann H, Yurasov S, Schaefer A, Young JW, Meffre E, Nussenzweig MC. Predominant autoantibody production by early human B cell precursors. Science. 2003;301:1374–1377. doi: 10.1126/science.1086907 12920303 10.1126/science.1086907

[9] Choo SY. The HLA system: genetics, immunology, clinical testing, and clinical implications. Yonsei medical journal. 2007 10.3349/ymj.2007.48.1.11 PMC2628004 17326240

[10] Pistillo MP, Mazzoleni O, Tanigaki N et al Human anti-HLA monoclonal antibodies: production, characterization, and application. Hum Immunol. 1988;21:265–278. 2836346 10.1016/0198-8859(88)90035-3

[11] Pistillo MP, Hammer J, Bono E et al A novel approach to human anti-HLA mABs production: use of phage display libraries. Hum Immunol. 1997;57:19–26. 9438191 10.1016/s0198-8859(97)00176-6

[12] Hunt DF, Henderson RA, Shabanowitz J et al Characterization of peptides bound to the class I MHC molecule HLA-A2.1 by mass spectrometry. Science. 1992;255:1261–1263. 1546328 10.1126/science.1546328

[13] Kasuga K. Comprehensive analysis of MHC ligands in clinical material by immunoaffinity-mass spectrometry. Methods Mol Biol. 2013;1023:203–218. doi: 10.1007/978-1-4614-7209-4_14 23765629 10.1007/978-1-4614-7209-4_14

[14] Battegay M, Fikes J, Di Bisceglie AM et al Patients with chronic hepatitis C have circulating cytotoxic T cells which recognize hepatitis C virus-encoded peptides binding to HLA-A2.1 molecules. J Virol. 1995;69:2462–2470. 7884894 10.1128/jvi.69.4.2462-2470.1995 PMC188921

[15] Wölfel T, Klehmann E, Müller C, Schütt KH, Meyer zum Büschenfelde KH, Knuth A. Lysis of human melanoma cells by autologous cytolytic T cell clones. Identification of human histocompatibility leukocyte antigen A2 as a restriction element for three different antigens. J Exp Med. 1989;170:797–810. 2788708 10.1084/jem.170.3.797 PMC2189434

[16] Kasper MR, Collins KL. Nef-mediated disruption of HLA-A2 transport to the cell surface in T cells. J Virol. 2003;77:3041–3049. doi: 10.1128/JVI.77.5.3041-3049.2003 12584329 10.1128/JVI.77.5.3041-3049.2003 PMC149742

[17] Lubben NB, Sahlender DA, Motley AM, Lehner PJ, Benaroch P, Robinson MS. HIV-1 Nef-induced down-regulation of MHC class I requires AP-1 and clathrin but not PACS-1 and is impeded by AP-2. Mol Biol Cell. 2007;18:3351–3365. doi: 10.1091/mbc.E07-03-0218 17581864 10.1091/mbc.E07-03-0218 PMC1951775

[18] Schumm M, Feuchtinger T, Pfeiffer M et al Flow cytometry with anti HLA-antibodies: a simple but highly sensitive method for monitoring chimerism and minimal residual disease after HLA-mismatched stem cell transplantation. Bone Marrow Transplant. 2007;39:767–773. doi: 10.1038/sj.bmt.1705676 17438586 10.1038/sj.bmt.1705676

[19] Choe W, Hwang MA, Jang S, Park CJ, Chi HS, Im HJ. Establishing a Population-Based HLA-Antibody Panel for Flow Cytometric Monitoring of Chimerism in HLA-Haploidentical Stem Cell Transplantation. Ann Clin Lab Sci. 2016;46:161–167. 27098622

[20] Kean LS, Singh K, Blazar BR, Larsen CP. Nonhuman primate transplant models finally evolve: detailed immunogenetic analysis creates new models and strengthens the old. Am J Transplant. 2012;12:812–819. doi: 10.1111/j.1600-6143.2011.03873.x 22177005 10.1111/j.1600-6143.2011.03873.x PMC3482466

[21] Glaze ER, Roy MJ, Dalrymple LW, Lanning LL. A Comparison of the Pathogenesis of Marburg Virus Disease in Humans and Nonhuman Primates and Evaluation of the Suitability of These Animal Models for Predicting Clinical Efficacy under the ‘Animal Rule’. Comp Med. 2015;65:241–259. 26141449 PMC4485633

[22] Scanga CA, Flynn JL. Modeling tuberculosis in nonhuman primates. Cold Spring Harb Perspect Med. 2014;4:a018564 doi: 10.1101/cshperspect.a018564 25213189 10.1101/cshperspect.a018564 PMC4292094

[23] Estep RD, Wong SW. Rhesus macaque rhadinovirus-associated disease. Curr Opin Virol. 2013;3:245–250. doi: 10.1016/j.coviro.2013.05.016 23747119 10.1016/j.coviro.2013.05.016 PMC3780577

[24] Schmitz JE, Korioth-Schmitz B. Immunopathogenesis of simian immunodeficiency virus infection in nonhuman primates. Curr Opin HIV AIDS. 2013;8:273–279. doi: 10.1097/COH.0b013e328361cf5b 23615117 10.1097/COH.0b013e328361cf5b PMC3857722

[25] Wiseman RW, Karl JA, Bohn PS, Nimityongskul FA, Starrett GJ, O’Connor DH. Haplessly hoping: macaque major histocompatibility complex made easy. ILAR J. 2013;54:196–210. doi: 10.1093/ilar/ilt036 24174442 10.1093/ilar/ilt036 PMC3814398

[26] Westbrook CJ, Karl JA, Wiseman RW et al No assembly required: Full-length MHC class I allele discovery by PacBio circular consensus sequencing. Hum Immunol. 2015;76:891–896. doi: 10.1016/j.humimm.2015.03.022 26028281 10.1016/j.humimm.2015.03.022

[27] Dudley DM, Karl JA, Creager HM, Bohn PS, Wiseman RW, O’Connor DH. Full-length novel MHC class I allele discovery by next-generation sequencing: two platforms are better than one. Immunogenetics. 2014;66:15–24. doi: 10.1007/s00251-013-0744-3 24241691 10.1007/s00251-013-0744-3 PMC3910708

[28] Budde ML, Wiseman RW, Karl JA, Hanczaruk B, Simen BB, O’Connor DH. Characterization of Mauritian cynomolgus macaque major histocompatibility complex class I haplotypes by high-resolution pyrosequencing. Immunogenetics. 2010;62:773–780. doi: 10.1007/s00251-010-0481-9 20882385 10.1007/s00251-010-0481-9 PMC3077881

[29] Karl JA, Graham ME, Wiseman RW et al Major histocompatibility complex haplotyping and long-amplicon allele discovery in cynomolgus macaques from Chinese breeding facilities. Immunogenetics. 2017;69:211–229. doi: 10.1007/s00251-017-0969-7 28078358 10.1007/s00251-017-0969-7 PMC5352482

[30] Leith JG, Clark DA, Matthews TJ et al Assessing human alloimmunization as a strategy for inducing HIV type 1 neutralizing anti-HLA responses. AIDS Res Hum Retroviruses. 2003;19:957–965. doi: 10.1089/088922203322588305 14678602 10.1089/088922203322588305

[31] Mowbray JF, Underwood JL, Michel M, Forbes PB, Beard RW. Immunisation with paternal lymphocytes in women with recurrent miscarriage.[letter]. Lancet 1987;2(8560):679–680. 2887954 10.1016/s0140-6736(87)92457-3

[32] Allen TM, Mothé BR, Sidney J et al CD8(+) lymphocytes from simian immunodeficiency virus-infected rhesus macaques recognize 14 different epitopes bound by the major histocompatibility complex class I molecule mamu-A*01: implications for vaccine design and testing. J Virol. 2001;75:738–749. doi: 10.1128/JVI.75.2.738-749.2001 11134287 10.1128/JVI.75.2.738-749.2001 PMC113970

[33] Shimizu Y, DeMars R. Production of human cells expressing individual transferred HLA-A,-B,-C genes using an HLA-A,-B,-C null human cell line. J Immunol. 1989;142:3320–3328. 2785140

[34] Barbas CF, Burton DR, Scott JK, Silverman GJ. Phage display: a laboratory manual. CSHL Press 2004

[35] Kuwata T, Katsumata Y, Takaki K, Miura T, Igarashi T. Isolation of potent neutralizing monoclonal antibodies from an SIV-Infected rhesus macaque by phage display. AIDS Res Hum Retroviruses. 2011;27:487–500. doi: 10.1089/aid.2010.0191 20854170 10.1089/AID.2010.0191

[36] Mothé BR, Horton H, Carter DK et al Dominance of CD8 responses specific for epitopes bound by a single major histocompatibility complex class I molecule during the acute phase of viral infection. J Virol. 2002;76:875–884. doi: 10.1128/JVI.76.2.875-884.2002 11752176 10.1128/JVI.76.2.875-884.2002 PMC136839

[37] Allen TM, Sidney J, del Guercio MF et al Characterization of the peptide binding motif of a rhesus MHC class I molecule (Mamu-A*01) that binds an immunodominant CTL epitope from simian immunodeficiency virus. J Immunol. 1998;160:6062–6071. 9637523

[38] Zhang ZQ, Fu TM, Casimiro DR et al Mamu-A*01 allele-mediated attenuation of disease progression in simian-human immunodeficiency virus infection. J Virol. 2002;76:12845–12854. doi: 10.1128/JVI.76.24.12845-12854.2002 12438610 10.1128/JVI.76.24.12845-12854.2002 PMC136722

[39] Egan MA, Kuroda MJ, Voss G et al Use of major histocompatibility complex class I/peptide/beta2M tetramers to quantitate CD8(+) cytotoxic T lymphocytes specific for dominant and nondominant viral epitopes in simian-human immunodeficiency virus-infected rhesus monkeys. J Virol. 1999;73:5466–5472. 10364294 10.1128/jvi.73.7.5466-5472.1999 PMC112603

[40] Kuroda MJ, Schmitz JE, Barouch DH et al Analysis of Gag-specific cytotoxic T lymphocytes in simian immunodeficiency virus-infected rhesus monkeys by cell staining with a tetrameric major histocompatibility complex class I-peptide complex. J Exp Med. 1998;187:1373–1381. 9565630 10.1084/jem.187.9.1373 PMC2212269

[41] Kling C, Steinmann J, Westphal E, Magez J, Kabelitz D. Adverse effects of intradermal allogeneic lymphocyte immunotherapy: acute reactions and role of autoimmunity. Hum Reprod. 2006;21:429–435. doi: 10.1093/humrep/dei316 16210388 10.1093/humrep/dei316

[42] Kwakkenbos MJ, van Helden PM, Beaumont T, Spits H. Stable long-term cultures of self-renewing B cells and their applications. Immunol Rev. 2016;270:65–77. doi: 10.1111/imr.12395 26864105 10.1111/imr.12395 PMC4755196

[43] de Groot NG, Otting N, Robinson J et al Nomenclature report on the major histocompatibility complex genes and alleles of Great Ape, Old and New World monkey species. Immunogenetics. 2012;64:615–631. doi: 10.1007/s00251-012-0617-1 22526602 10.1007/s00251-012-0617-1 PMC3396756

[44] De Vito LD, Mason BP, Jankowska-Gan E et al Epitope fine specificity of human anti-HLA-A2 antibodies. Identification of four epitopes including a haptenlike epitope on HLA-A2 at lysine 127. Hum Immunol. 1993;37:165–177. 7503972 10.1016/0198-8859(93)90182-z

[45] El-Awar NR, Akaza T, Terasaki PI, Nguyen A. Human leukocyte antigen class I epitopes: update to 103 total epitopes, including the C locus. Transplantation. 2007;84:532–540. doi: 10.1097/01.tp.0000278721.97037.1e 17713439 10.1097/01.tp.0000278721.97037.1e

[46] Avril A, Froude JW, Mathieu J, Pelat T, Thullier P. Isolation of antibodies from non-human primates for clinical use. Curr Drug Discov Technol. 2014;11:20–27. 23410051 10.2174/15701638113109990030

[47] Mudd PA, Watkins DI. Understanding animal models of elite control: windows on effective immune responses against immunodeficiency viruses. Curr Opin HIV AIDS. 2011;6:197–201. doi: 10.1097/COH.0b013e3283453e16 21502922 10.1097/COH.0b013e3283453e16 PMC3789597

[48] Larsen CP, Page A, Linzie KH et al An MHC-defined primate model reveals significant rejection of bone marrow after mixed chimerism induction despite full MHC matching. Am J Transplant. 2010;10:2396–2409. doi: 10.1111/j.1600-6143.2010.03272.x 20849552 10.1111/j.1600-6143.2010.03272.x PMC2980834

[49] Kean LS, Adams AB, Strobert E et al Induction of chimerism in rhesus macaques through stem cell transplant and costimulation blockade-based immunosuppression. Am J Transplant. 2007;7:320–335. doi: 10.1111/j.1600-6143.2006.01622.x 17241112 10.1111/j.1600-6143.2006.01622.x

[50] Lau M, Vayntrub T, Grumet FC et al Short tandem repeat analysis to monitor chimerism in macaca fascicularis. Am J Transplant. 2004;4:1543–1548. doi: 10.1111/j.1600-6143.2004.00529.x 15307845 10.1111/j.1600-6143.2004.00529.x

[51] Kim BH, Jang S, Lee Y, Park N, Cho YU, Park CJ. Lineage-Specific Chimerism Monitoring Using Flow Cytometry with Anti HLA-Antibodies in Haploidentical Hematopoietic Stem Cell Transplantation. Blood. 2014

[52] Drabbels JJ, van de Keur C, Kemps BM et al HLA-targeted flow cytometric sorting of blood cells allows separation of pure and viable microchimeric cell populations. Blood. 2011;118:e149–55. doi: 10.1182/blood-2011-06-362053 21931111 10.1182/blood-2011-06-362053

